# Persistent asymptomatic bacteriuria despite targeted antibiotic treatment predicts infectious complications after RIRS: a 10-year experience with 1122 procedures

**DOI:** 10.1007/s00345-026-06412-2

**Published:** 2026-05-19

**Authors:** Andrea Bosio, Eugenio Alessandria, Glauco Bertello, Claudia Gozzo, Luca Micai, Eugenia Vercelli, Edoardo Ossella, Giulia Sanfilippo, Alessandro Bisconti, Fabrizio Fop, Paolo Gontero

**Affiliations:** 1 Department of Surgical Sciences, University of Turin, Turin, Italy; 2 Department of Urology AOU Città della Salute e della Scienza, Molinette University Hospital, Turin, Italy; 3 Department of Nephrology, AOU Città della Salute e della Scienza, Molinette University Hospital, Turin, Italy

**Keywords:** Urolithiasis, Ureteroscopy, RIRS, Urine culture, Infectious complications

## Abstract

**Purpose:**

To evaluate the impact of persistently positive preoperative urine culture (pUC) despite tergeted antibiotic treatment on infectious complications (IC) after RIRS for stone treatment.

**Methods:**

Prospectively collected data from Institution RIRS database (Città della Salute e della Scienza, Molinette University Hospital, Turin, Italy) were analyzed - June 2014 to December 2024. Negative culture (nUC) received single-dose prophylaxis; pUC received targeted therapy and repeat culture. Persistent pUC underwent RIRS under antibiotic coverage. IC within 30 days were recorded. Descriptive statistics and multivariable logistic regression were performed.

**Results:**

Among the 1,122 RIRS included, peristent pUC was found in 19.6% (220/1,122). Overall IC rate was 8.9% (100), including minor IC (Clavien I–II: 8.5%, *n* = 93), urosepsis (IVb: 0.5%, *n* = 6), and one death due to septic shock (V). IC were significantly more frequent in pUC patients (13.2% vs. 8.9%, *p* = 0.01). pUC group had significantly more females (62.6% vs. 34.1%), higher age (60.5 ± 15.7 vs. 55.8 ± 13.6 years), larger stones (12.7 ± 6.3 vs. 11.6 ± 5.2 mm) and higher comorbidity (CCI ≥ 2: 65.2% vs. 57.1%) – all *p* < 0.05. On multivariable analysis, pUC (OR 1.80, 95% CI 1.06–3.08, *p* = 0.03) and female sex (OR 1.67, 95% CI 1.02–2.75, *p* = 0.04) resulted independent predictors of IC. Limitations include the observational single-center design.

**Conclusions:**

Persistent asymptomatic bacteriuria increases the risk of postoperative IC after RIRS, despite guideline-based targeted therapy. Careful perioperative management, particularly in pUC and female patients, is essential to limit IC.

## Introduction

Retrograde intrarenal surgery (RIRS) is a widely adopted, minimally invasive technique for the treatment of renal stones, offering high success rates and low morbidity [1]. Most reported complications are minor (Clavien–Dindo grades I–II) [1, 2]. Postoperative infections currently represent one of the most frequent complications following endourological stone management, with variable clinical presentations ranging from postoperative fever and urinary tract infection (UTI) to urosepsis, posing a risk of severe outcomes [3, 4]. Historically, sepsis was defined as an infection accompanied by at least two Systemic Inflammatory Response Syndrome (SIRS) criteria - abnormal temperature, heart rate, respiratory rate, or white blood cell count [5]. The more recent Sepsis-3 definition describes sepsis as “life-threatening organ dysfunction caused by a dysregulated host response to infection” [6]. Identifying preoperative risk factors is therefore crucial to improving patient safety and outcomes. Several studies have evaluated infectious complications (IC) following ureteroscopy [7–9], and a preoperative positive urine culture (pUC) has emerged as a major concern in their prevention [7, 8, 10]. Significant asymptomatic bacteriuria has long been associated with an increased risk of IC after RIRS and other endourological procedures, and current guidelines recommend screening for and treating pUC before surgery [1, 11]. However, the optimal management of patients with persistent pUC remains debated, as supporting evidence is limited and clinical practice varies widely [1, 12, 13].

This study aimed to evaluate the impact of preoperative pUC on the incidence of postoperative IC following RIRS. By analysing prospectively collected data from our Stone Centre, we seek to determine whether persistent preoperative asymptomatic bacteriuria despite targeted antibiotic treatment represents a risk factor for early postoperative infectious events, along with other potentially relevant clinical variables.

## Materials and methods

All patients who underwent RIRS for kidney or upper ureteral stones at our Stone Centre (AOU Città della Salute e della Scienza, Molinette University Hospital, Turin, Italy) from June 2014 onwards were asked to provide written consent for the collection of clinical data. Data were collected in our Institution prospectively maintained RIRS database, which was registered as internal audit of our Department. The most recent case included in the analysis underwent RIRS in December 2024. Exclusion criteria were: age < 18 years, pregnancy, unsuccessful access to the upper urinary tract, missing preoperative urine culture data due to urgent procedures, procedures combined with a percutaneous approach, bilateral procedures, urinary diversion, or anatomical abnormalities.

Postoperative complications within 30 days were classified using the Modified Clavien Classification System (MCCS) for RIRS [14], focusing on IC: minor (grades I–II, including fever and UTI); grade IVb (urosepsis, defined according to Sepsis-3), requiring transfer to the intensive care unit; and grade V (death related to septic shock). All information was recorded during hospitalisation and follow-up visits. The study was conducted in accordance with the principles of the Declaration of Helsinki, and written informed consent was obtained from all participants. The STROBE checklist for observational cohort studies was followed.

### Preoperative assessment

Urinalysis and urine culture were performed as part of the preoperative evaluation. In patients with a negative urine culture (nUC), a single dose of prophylactic antibiotics was administered intravenously at induction of anaesthesia, according to the EAU Guidelines on Urological Infections—trimethoprim–sulfamethoxazole, a second- or third-generation cephalosporin, or an aminopenicillin combined with a beta-lactamase inhibitor [11].

If the urine culture was positive for aymptomatic bacteriuria, targeted antibiotic therapy was prescribed, and a repeat urine culture was performed prior to surgery. In cases of persistent pUC, patients underwent RIRS under perioperative antibiotic coverage, usually began 2–3 days before intervention and continued afterwards. For multi-drug-resistant (MDR) organisms or equivocal results, an infectious disease consultation was obtained before the procedure.

### RIRS procedure

After standardised antiseptic preparation, semirigid ureteroscopy was first performed to assess the ureter. A ureteral access sheath (9.5/11.5–10.7/12.7 Fr, Flexor, Cook, USA; 11/13 Fr, Navigator, Boston Scientific, USA) was then introduced. RIRS was performed using a Flex-X2 (Karl Storz, Germany), Viper (Richard Wolf, USA), or Uscope 3022 (Pusen, China) flexible ureterorenoscope. Holmium laser lithotripsy was performed with a 30 W Holmium laser (Rocamed, Monaco) using dusting settings, followed by the popcorn technique and retrieval of residual fragments with a basket. Placement of a ureteral stent at the end of the procedure was left to the surgeon’s discretion.

### Follow-up

The first follow-up visit was typically scheduled two to three months after treatment and included radiological imaging, as clinically indicated. Any complications were recorded during unscheduled consultations or follow-up visits. The procedure was considered stone-free in case of residual fragments ≤ 4 mm.

### Evaluation criteria

The primary outcome of this study was to assess the association between preoperative pUC and the incidence of early (within 30 days) postoperative IC following RIRS. Secondary outcomes included the identification of other potential factors associated with postoperative IC.

### Statistical analysis

Descriptive statistics were used to summarise patient demographics, stone characteristics, operative variables, and postoperative outcomes. Continuous variables were expressed as means ± standard deviations (SD) or medians with interquartile ranges (IQR), as appropriate, while categorical variables were reported as frequencies and percentages. Comparisons between the pUC and nUC groups were performed using the Student’s t-test or Mann–Whitney U test for continuous variables, and the Pearson’s chi-square test or Fisher’s exact test for categorical variables, as appropriate. A multivariable logistic regression model was performed. Covariates for the model were included based on a significance level of *p* < 0.1 in univariable preliminary analysis, and by their clinical relevance according to previous literature. An interaction term between two associated covariates, previously identified through bivariate analysis, was added. All statistical tests were two-sided, and a p-value < 0.05 was considered statistically significant. Based on preliminary internal audits, the prevalence of complications was estimated at 10%. Given that the rate of positive urine cultures was 18% among patients without complications and increased to approximately 30% among those who developed complications, a power analysis was conducted for a Chi-squared test of proportions. Using a significance level (α) of 0.05, a power (1-β) of 0.80, and a group allocation ratio of 0.1, the calculated sample size was 1012 patients for the group without complications and 102 patients for the group with complications. The prospective database was reviewed by an independent analyst (FF) responsible for statistical analysis. Data were recorded in a Microsoft Access database, and all analyses were performed using IBM SPSS Statistics for Windows, Version 29.0.2.0 (IBM Corp., Armonk, NY, USA).

## Results

**Fig. 1:**
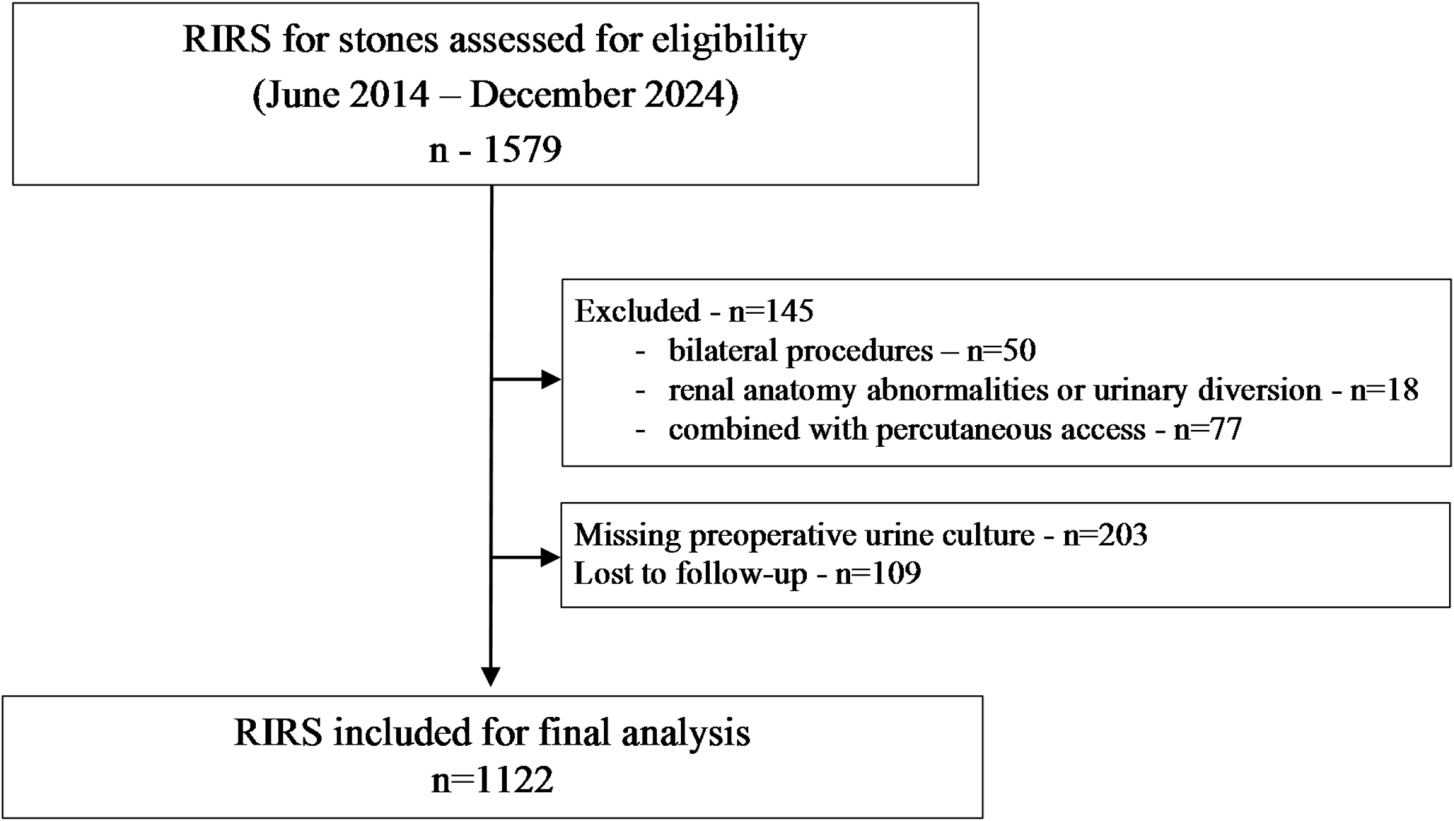
STROBE flow-chart

A total of 1,122 RIRS procedures were included in the final analysis (Fig. 1 – STROBE flowchart). Most patients were male (60.3%, 677/1122), with a mean age of 56.7 ± 14.2 years and a mean BMI of 26.2 ± 4.9 kg/m². The mean stone size was 11.9 ± 5.4 mm, and the median stone volume was 493 mm³ [203–936]. Stones were predominantly located in the lower calyces (33%, 370/1122) and renal pelvis (23%, 258/1122). A preoperative ureteral stent was present in 39% (438/1122) of cases, and the median operative time was 75 min [50–90]. The SFR was 81.2% (911/1122).

Preoperative urine culture was persistently positive in 19.6% (220/1122) of cases, while the remaining 80.4% (902/1122) had nUC. MDR organisms were identified in 24.5% of pUC cases (54/220), the vast majority due to extended-spectrum β-lactamase (ESBL) production (19.5%, 43/220). Baseline characteristics are reported in Table 1.

### Infectious complications

The overall rate of IC was 8.9% (100/1122): minor complications (grades I–II, including fever and UTI) occurred in 8.5% (93/1122), while urosepsis (grade IVb) was reported in 0.5% (6/1122). One death occurred in the pUC group due to septic shock. Infectious complications were significantly more frequent in the pUC group, both overall (13.2% vs. 8.9%, *p* = 0.01) and for minor complications (grades I–II) (11.9% vs. 7.4%, *p* = 0.03). Postoperative infectious complications according to the MCCS are reported in Table 2. The pUC and nUC groups were comparable in terms of BMI, diabetes, preoperative stenting, stone location, operative time, and length of stay. Descriptive statistics showed a significantly higher proportion of female patients in the pUC group (62.6% vs. 34.1%, *p* < 0.01), higher mean age (60.5 ± 15.7 vs. 55.8 ± 13.6 years, *p* < 0.01), larger mean stone size (12.7 ± 6.3 vs. 11.6 ± 5.2 mm, *p* < 0.01), and a significantly higher proportion of patients with CCI ≥ 2 (65,2% vs. 57,1%, *p* = 0.04). Descriptive statistics for pUC and nUC groups are reported in Table 3.

### Predictors of infectious complications

Potentially relevant variables (sex, pUC, CCI, stone size > 10 mm, operative time > 65 min and preoperative stent) were included in the multivariable logistic regression model. Multivariable analysis identified pUC (OR 1.80, 95% CI 1.06–3.08, *p* = 0.03) and female sex (OR 1.67, 95% CI 1.02–2.75, *p* = 0.04) as independent predictors for postoperative IC. Univariable and multivariable analysis results are presented in Table 4.

## Discussion

Our results demonstrate that persistent asymptomatic bacteriuria is significantly associated with a higher rate of early postoperative IC following RIRS, despite guideline-based targeted preoperative and perioperative antibiotic therapy. Although most IC were minor (8.5%, Clavien I–II), the occurrence of cases of urosepsis (0.5%) and one death related to sepsis underscores the potential severity of these events. On multivariable analysis, both pUC and female sex emerged as independent predictors of IC, confirming their key role in postoperative infectious risk. Recent systematic reviews have provided complementary evidence on the multifactorial nature of infection risk after RIRS. Dybowski et al. reported IC rates ranging from 2.8% to 7.5% (mean 7.1%), identifying seven independent risk factors, including prolonged operative time and pUC [8]. Similarly, the systematic review by Corrales et al. found sepsis rates ranging from 0.5% to 11.1% and septic shock rates from 0.3% to 4.6%, with independent predictors including stone size, female sex, pUC, longer operative time, and diabetes mellitus [7]. Bhojani et al. reported an even higher urosepsis rate (5%), identifying preoperative stent placement, pUC, and longer procedure duration among the associated factors [15]. Our results report that a persistent positive preoperative urine culture and female sex are significant predictors of IC. Female patients are particularly susceptible to postoperative infection due to anatomical and physiological predispositions, and several studies have consistently shown higher rates of IC in women undergoing endourological procedures [7, 8].

The near-systematic use of a ureteral access sheath, reflecting our current surgical practice at our center, provides a high degree of homogeneity throughout the study population.

Despite the relationship between UAS use and postoperative infections remains debated, the use of ureteral access sheath may have had a beneficial influence on the risk of life-threatening IC [10, 16].

As stated in the EAU Guidelines, preoperative pUC should always be treated before surgery; however, evidence supporting specific preventive strategies remains limited. The American Urological Association (AUA) likewise recommends treating pUC prior to surgery, with perioperative antibiotic prophylaxis guided primarily by prior urine culture results, local antibiograms, and the current Best Practice Policy Statement on Urologic Surgery Antibiotic Prophylaxis [17, 18]. Despite these recommendations, the best timing, duration, and choice of antibiotic therapy remain subjects of ongoing debate. The heterogeneity of patient populations, stone burden, and surgical techniques makes it challenging to define a universal protocol. Evidence suggests that while a single perioperative antibiotic dose may be sufficient for patients with sterile urine, targeted, culture-based strategies are preferable in high-risk patients with positive preoperative cultures [12, 13, 19, 20]. Whenever feasible, the procedure should be postponed until a negative urine culture is obtained. However, as recognized by the AUA Best Practices, delaying surgery is not always safe and may expose patients with persistent stone disease to additional risks of recurrent infection and sepsis [18]. Urologists should therefore remain aware of local bacterial resistance patterns [21] and make every effort to adhere to international recommendations. Nonetheless, adherence seems to be suboptimal, with a reported overall compliance with AUA Best Practice guidelines has been reported at less than 60% for urological procedures [22]. Importantly, unnecessary antibiotic use should be avoided to prevent the development of antimicrobial resistance, favoring instead individualized antibiotic strategies when appropriate.

### Strengths and limitations

The findings of this study are based on a large, prospectively maintained RIRS database, including standardized perioperative protocols and more than 1,000 procedures. The homogeneity of surgical technique and perioperative management is a major strength, with consistent antibiotic prophylaxis in patients with negative urine cultures, standardized pUC management, consisting in targeted therapy, UC repeat, and RIRS under perioperative antibiotic coverage, in cases of persistent pUC.

Limitations exist, primarily due to the observational and single-centre nature of the study. Data were collected prospectively, but the analysis was performed retrospectively. Our study focused on a single academic medical institution, which may limit generalisability. 312 patients were excluded due to missing preoperative urine culture data or incomplete follow-up. This may have introduced a degree of selection bias, despite the large final cohort included in the analysis. Moreover, potentially valuable tools, such as stone culture and intrarenal urine culture, were not assessed due to limited data availability [23]. However, these findings have led us to systematically perform intraoperative urine cultures in order to obtain a baseline result at the start of the procedure. Despite these limitations, this large cohort of patients from a regional referral center provides meaningful real-world data that enrich the current evidence and may stimulate discussion on a clinically relevant yet underexplored issue, particularly given the growing impact of antimicrobial resistance in modern medicine.

**Table 1:** Patients demographic, clinical and operative data of the study cohort (n – 1122)

Study population	n - 1122
Age (years) mean (SD)	56.7 + 14.2
Male - female [% (n)]	60.3% (677)–39.7% (445)
Right - left [% (n)]	45% (505)− 55% (617)
BMI (kg/m^2^) mean (SD)	26.2 ± 4.9
Charlson Comorbidity Index - CCI [% (n)]	
- 0	20.2% (227)
- 1	21.1% (237)
- ≥ 2	58.7% (658)
Preoperative stent [% (n)]	39% (438)
Main stone size (mm) mean (SD)	11.9 ± 5.4
Stone volume (mm^3^) median [IQR]	493 [203–936]
Stone density (Hounsfield Unit) median [IQR]	900 [509, 1200]
Location [% (n)]	
- Renal pelvis	23% (258)
- Superior calyx	9.4% (105)
- Medium calyx	13.8% (155)
- Inferior calyx	33% (370)
- UPJ	8.8% (99)
- Upper ureter	12% (135)
SFR [% (n)]	81.2% (807)
Operative time (min) median [IQR]	75 [50, 90]
Hospitalization (days) median [IQR]	1 [1, 2]
Use of UAS [% (n)]	95.2% (1068)
Postoperative stent [% (n)]	94.3% (1058)
Further treatment [% (n)]	8.3% (93)

**Table 2:** RIRS postoperative infectious complications according to MSSC, and based on urine culture (n – 1122)

Infectious complications		*n* = 1122	Positive UC *n* = 220 19.6%	Negative UC *n* = 902 80.4%	*p*-value
Overall		100/1122 8.9%	29/220 13.2%	70/902 7.8%	**0.01**
I and II - minor	Fever and UTI	93/1122 8.5%	26/220 11.9%	67/902 7.4%	**0.03**
IVb	Urosepis	6/1122 0.5%	4/220 1.8%	2/902 0.2%	0.39
V	Death	1	0	1	

*UC* urine culture, *UTI* urinary tract infection, statistically significant p-values are reported in bold

**Table 3:** Descriptive statistics based on positive and negative preoperative urine culture (n – 1122)

	Positive UC *n* = 219 19.5%	Negative UC *n* = 903 80.5%	*p*-value
**Patients’ characteristics**			
Age (years) mean (SD)	60.5 ± 15.7	55.8 ± 13.6	**< 0.01**
Male/female [% (n)]	37.4% (82/219) 62.6% (137/219)	65.9% (595/903) 34.1% (308/903)	**< 0.01**
BMI (kg/m^2^) mean (SD)	26.09 ± 5.51	26.21 ± 4.81	0.18
CCI ≥ 2 [% (n)]	65.3% (143/219)	57.1% (516/903)	**0.04**
Diabetes [% (n)]	10.5% (23/219)	10.9% (98/903)	0.55
Preoperative stent [% (n)]	42.5% (93/219)	38.3% (346/903)	0.51
**Stone**			
Main stone size (mm) mean (SD)	12.7 ± 6.3	11.6 ± 5.2	**< 0.01**
***Location***			
Ureteral and UPJ [% (n)]	17% (37/219)	22.7% (205/903)	0.07
Pyelocaliceal [% (n)]	83% (182/219)	77.3% (698/903)	
**RIRS**			
Operative time (min) mean (SD)	68 ± 30	68 ± 30	0.94
Length of stay (days) mean (SD)	1 [1, 2]	1 [1, 2]	0.85

*CCI* Charlson Comorbidity Index, *UC* urine culture, *UPJ* ureteropelvic junction, statistically significant p-values are reported in bold

**Table 4:** Univariable and multivariable analysis predicting postoperative infectious complications after RIRS (n – 1122)

	UVA model OR, 95% CI *p* value	MVA model OR, 95% CI *p* value
Positive urine culture	1.79, 1.13–2.83 *p* = 0.01	1.80, 1.06–3.08 *p* = 0.03
Female sex	1.89, 1.25–2.86 *p*< 0.01	1.67, 1.02–2.75 *p* = 0.04
CCI ≥ 2	1.45, 0.9–2.33 *p* = 0.13	1.42, 0.87–2.34 *p* = 0.16
Stone size > 10 mm	0.84, 0.55-1.26 *p* = 0.39	0.78, 0.48–1.27 *p* = 0.31
Operative time > 65 min	0.97, 0.65-1.48 *p* = 0.92	0.95, 0.58–1.54 *p* = 0.83
Preoperative stent	0.98, 0.64-1.51 *p* = 0.95	0.97, 0.59–1.58 *p* = 0.91

*CCI* Charlson Comorbidity Index, *UVA* univariable model, *MVA* multivariable model

## Conclusions

In this cohort of 1,122 RIRS for stone disease, the overall incidence of infectious complications was 8.9%. A persistent positive preoperative asyntomatic bacteriuria was significantly associated with a higher rate of early infectious events, despite targeted guideline-based preoperative, and perioperative antimicrobial management. Both preoperative positive urine culture and female sex resulted independent predictors of postoperative infection. Careful preoperative assessment and perioperative management of peristent pUC may therefore play a key role in trying to limit infectious complications after RIRS.

## Data Availability

The findings of this study are available from Institution RIRS database (AOU Città della Salute e della Scienza, Molinette University Hospital-Turin, Dept. of Urology), available upon request.

## Abbreviations

IC: Infectious complications
nUC: Negative urine culture
pUC: Positive urine culture
MCCS: Modified clavien classification system
MDR: Multi-drug-resistant
MVA: Multivariable logistic regression analysis
RIRS: Retrograde intrarenal surgery
SIRS: Systemic inflammatory response syndrome
UTI: Urinary tract infection
UVA: Ablelogistic regression analysis
